# Neuromyelitis Optica Spectrum Disorder Resembling Wernicke’s Encephalopathy: A Case Report and Review of the Literature

**DOI:** 10.1177/19418744241228004

**Published:** 2024-01-16

**Authors:** Sloan Lynch, Nil Saez Calveras, Anik Amin

**Affiliations:** 1 12334 University of Texas Southwestern Medical Center, Dallas, TX, USA; 2 Parkland Health, Dallas, TX, USA

**Keywords:** neuromyelitis optica, demyelinating diseases, wernicke encephalopathy, imaging, case report

## Abstract

We describe a case of Neuromyelitis Optica Spectrum Disorder (NMOSD) mimicking Wernicke’s Encephalopathy (WE) to highlight an atypical presentation of NMOSD. A 39-year-old female presented with subacute encephalopathy and progressive ophthalmoplegia. Her MRI revealed T2 hyperintensities involving the mammillary bodies, periaqueductal grey matter, medial thalami, third ventricle, and area postrema. Whole blood thiamine levels were elevated and she did not improve with IV thiamine. CSF was notable for lymphocytic pleocytosis and elevated protein. She tested positive for serum Aquaporin-4 (AQP4) antibody. Subsequent imaging revealed multilevel lesions in the cervical and thoracic spinal cord. Her CSF GFAP antibody also came back positive. She steadily and significantly improved after high-dose IV steroids and plasmapheresis. She later started on chronic rituximab therapy. This represents a unique case of NMOSD presenting with the classical clinical and imaging features of WE, as opposed to the typical presenting symptoms of NMOSD. As such, demyelinating disorders should be considered when there is concern for diencephalic and midline pathologies, particularly without classic WE risk factors. Conversely, clinicians should be aware of secondary nutritional complications arising from severe area postrema syndrome.

## Case Report

A 39-year-old female with no significant past medical history presented to Parkland Hospital due to progressive lethargy. The patient was unable to provide history due to somnolence, but she lived with multiple family members who noticed symptoms starting 3 weeks prior. Her initial symptoms included fatigue and forgetfulness. Shortly thereafter, the patient was unable to keep her eyes open when engaging in conversations, which the family attributed to sleepiness. Her somnolence progressed to falling asleep mid-meal and eventually inability to leave her bed.

**Figure 1.**
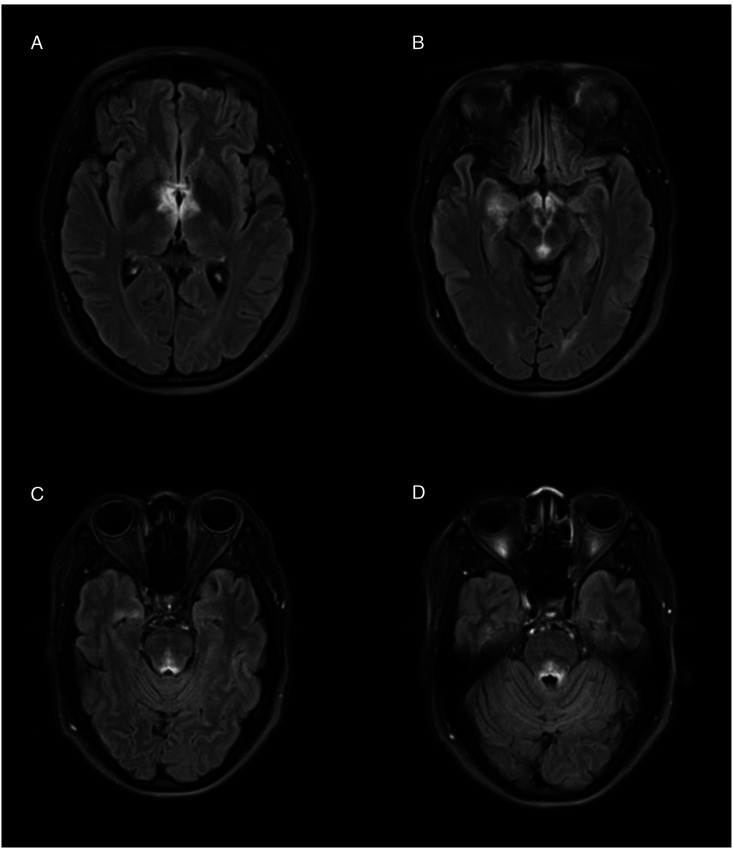
Patient’s initial T2 FLAIR MR brain imaging findings. (A) Involvement of the bilateral medial thalami and area surrounding the third ventricle. (B) Involvement of the bilateral mammillary bodies, periaqueductal grey matter and right medial temporal lobe. (C, D) Longitudinal extension with involvement of the fourth ventricle.

Upon presentation to the emergency department, the patient’s exam was notable for somnolence, bilateral ptosis, and impaired extraocular movements consisting of absent adduction and restricted movement in all other directions bilaterally. An MRI brain and orbits with and without contrast was remarkable for extensive non-enhancing T2 hyperintensities involving the periaqueductal grey, medial thalami, mammillary bodies, surrounding third ventricle, and area postrema. There was no involvement of the optic nerves (Figure 1). This presentation was concerning for Wernicke’s encephalopathy, so whole blood thiamine levels were promptly drawn and high-dose IV thiamine was started. However, family members denied any suspected alcohol use disorder or history of bariatric surgery. They reported she ate regular, full meals without dietary restrictions. As such, the differential diagnosis was broadened to include arbovirus infection and demyelinating disease.

At this time, serum Aquaporin-4 (AQP4) and Myelin Oligodendrocyte Glycoprotein (MOG) antibodies were collected. The patient was started on empiric IV acyclovir, and a lumbar puncture was performed. Initial CSF studies were notable for 50 nucleated cells with a 95% lymphocytic predominance, protein 83 mg/dL, normal glucose, and negative HSV PCR. IV acyclovir was discontinued. Despite the empiric thiamine the patient continued to decline. Her extraocular movements progressed to complete ophthalmoplegia, she developed recurrent hiccupping, her somnolence worsened, and she developed respiratory complications. She was transferred to the Neurocritical Care Unit by hospital day 6.

**Figure 2.**
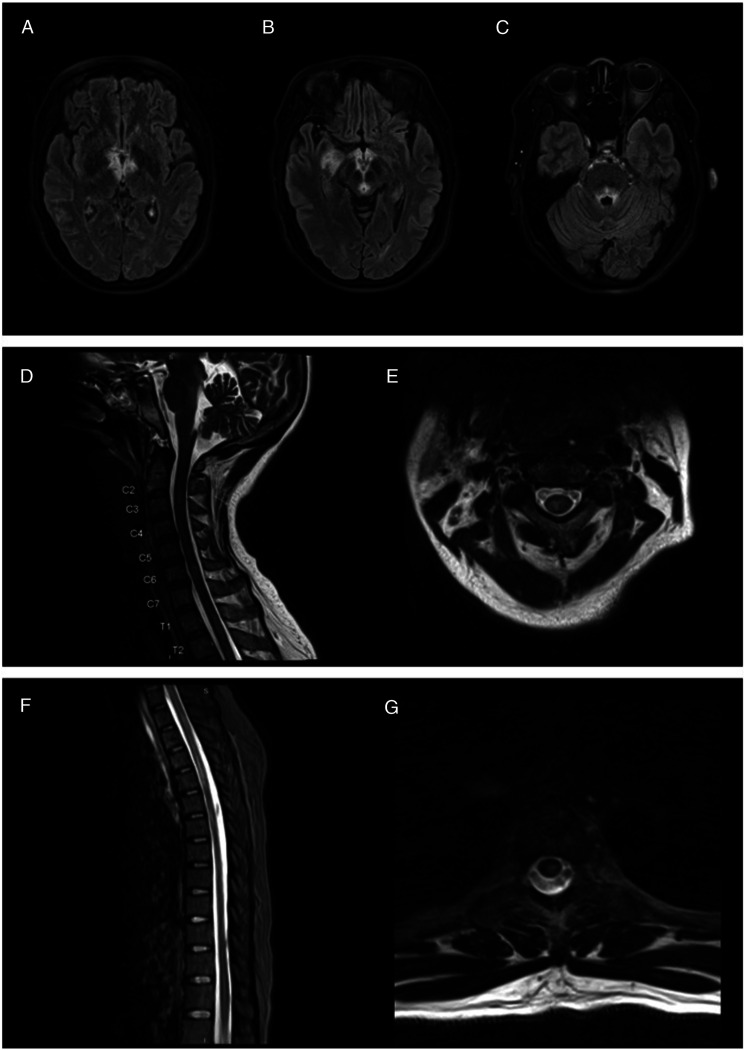
Patient’s repeat MR brain + MR cord imaging findings. (A) Bilateral medial thalami involvement with increased signal in the right thalamus. (B) Involvement of the bilateral mammillary bodies, periaqueductal grey matter, and increased signal in the right anterior temporal lobe. (C) Involvement of the fourth ventricle. (D)**:** Sagittal view of multilevel periventricular hyperintense lesions in the cervical cord (1-2 vertebral bodies long). (E) Axial view of a periventricular hyperintense lesion in the cervical spinal cord. (F) Sagittal view of multilevel periventricular hyperintense lesions in the thoracic cord (1-4 vertebral bodies long). (G) Axial view of a periventricular hyperintense lesion in the thoracic spinal cord.

Meanwhile, her serum and CSF West Nile Virus (WNV) IgG antibodies came back positive, while the IgM antibodies came back negative. In collaboration with infectious disease specialists, we favored this result to be more consistent with prior flavivirus exposure (patient previously lived in Burma) than acute WNV encephalitis. Additionally, whole blood thiamine level returned elevated at 191 nanomoles per liter. On hospital day 8, the patient’s serum AQP4-IgG titer returned positive at 1:80, and she was diagnosed with NMOSD. Therefore, she was started on a 5-day course of high dose IV methylprednisolone (1 gram daily) alongside a 7-round course of plasmapheresis. MRIs of her brain and spine taken 10 days after the initial MR images were remarkable for multiple non-enhancing T2 hyperintensities at various spinal levels in the cervical and thoracic cord (Figure 2). Finally, the patient’s autoimmune/paraneoplastic CSF studies returned with a positive glial fibrillary acidic protein (GFAP) titer at 1:32; however, the serum GFAP titer was negative.

The patient’s mental and overall status gradually began to improve with these treatments, although her hospital course was complicated by persistent dysphagia requiring G-tube placement. The patient received her first dose of rituximab while in the hospital and was discharged on hospital day 45 to an inpatient rehabilitation facility. She achieved significant improvement in mental status and extraocular movements. She has since continued to follow with the neurology clinic where she receives rituximab infusions every 6 months. Her symptoms have resolved except for persistent fatigue and dizziness. Her G-tube has been removed.

## Discussion

NMOSD is an autoimmune neurological disease typically caused by IgG antibodies against Aquaporin 4 (AQP4-IgG), usually manifesting as optic neuritis or transverse myelitis.^1-3^ However, demyelination in other CNS regions can lead to other presentations, such as area postrema lesions causing intractable nausea, vomiting, and hiccups. Encephalopathy, however, is considered atypical in NMOSD. It is more frequently seen in young children with MOG antibody-associated disease where it can manifest with altered mental status and seizures.^3^

Wernicke’s encephalopathy is a well-described complication of vitamin B1 deficiency and can present with encephalopathy symptoms, oculomotor dysfunction, and ataxia with gait imbalance. Imaging findings include hyperintensities of the bilateral medial thalami, periaqueductal gray area, and midbrain tectum on T2-weighted MRI.^4^ Common causes of this condition include alcohol abuse and GI absorption defects.^5^

This case is unique in the way it resembles both the clinical and radiographic phenotype of Wernicke’s encephalopathy, but without traditional risk factors for this condition or proven thiamine deficiency. Involvement of the area postrema in NMOSD can lead to persistent vomiting which can trigger nutritional deficiencies. Two prior case reports have described the development of Wernicke’s encephalopathy following the diagnosis of NMOSD.^6,7^ However, in our case the thiamine level drawn prior to supplementation was elevated, and the patient did not improve with high dose thiamine. Three prior case reports and a case series have raised the possibility of NMOSD mimicking the features of Wernicke’s encephalopathy.^8-11^ However, all but one of these cases identified a potential etiology for thiamine deficiency (most commonly prolonged vomiting), and none of these cases could exclude secondary WE because thiamine levels were not collected. Furthermore, in these cases either mammillary body involvement was not present or a more classic NMOSD presentation (eg, optic neuritis) was also present. One of the remarkable findings in our case was how well both her symptoms (progressive encephalopathy and ophthalmoplegia) and imaging findings (including bilateral mammillary body involvement) matched WE. Only the subtler imaging findings of ventricular surface and area postrema involvement prompted us to order the serum AQP4 antibody test (considered less likely at time of order). Of note, many of the areas affected in WE (eg, medial thalami and periaqueductal grey), are enriched in AQP4 due to their periventricular location. This likely explains the radiographic similarity between our NMOSD case and WE.

The positive CSF GFAP antibody in this case is of uncertain significance. GFAP autoantibodies have been described in GFAP astrocytopathy, which usually manifests as an acute to subacute meningoencephalomyelitis and can be associated with longitudinally-extensive T2 spinal cord hyperintensities.^12^ Although this clinical presentation would fit with our patient’s course and could explain her spinal cord lesions, extraocular eye movement impairment is uncommon in GFAP astrocytopathy. This condition is also associated with periventricular radial linear or meningeal enhancement, which were not seen on this patient’s imaging.^12^ On the other hand, this antibody has been observed in the serum of patients with traumatic brain injuries, suggesting its presence may represent a marker of CNS injury rather than causative pathology.^13^ Furthermore, there is increasing evidence to suggest that an elevated GFAP antibody titer is associated with antibody positive NMOSD, and that GFAP titers may serve as biomarkers for NMOSD.^14,15^ Therefore, the positive CSF GFAP in this case is likely an expression of astrocyte damage due to NMOSD rather than primary GFAP astrocytopathy.

Overall, this case further supports the notion that NMOSD can exhibit a diverse range of symptoms and imaging features, occasionally mimicking other neurological diseases. In our case, there was a remarkable resemblance to Wernicke’s encephalopathy, so much that the possibility of NMOSD area postrema syndrome triggering a Wernicke’s encephalopathy cannot be fully excluded. The significance of a concurrently positive CSF GFAP antibody in NMOSD cases also warrants further investigation. A high index of suspicion can promote prompter treatment and hence better outcomes in atypical NMOSD cases.
